# Precision engineering of upstream open reading frames for translational regulation and crop improvement

**DOI:** 10.3389/fgeed.2026.1818972

**Published:** 2026-09-21

**Authors:** Linjie Xu, Jiaojiao Ren, Jinjie Zhu

**Affiliations:** 1 College of Agronomy, Xinjiang Agricultural University, Urumqi, Xinjiang, China; 2 State Key Laboratory of Crop Gene Resources and Breeding, Institute of Crop Sciences, Chinese Academy of Agricultural Sciences, Beijing, China

**Keywords:** crop improvement, fine-tuning, genome editing, translational control, uORFs

## Abstract

Upstream open reading frames (uORFs) are pervasive cis-regulatory elements in the 5′untranslated regions (UTRs) of eukaryotic mRNAs that modulate translation of downstream main open reading frames (mORFs) by shaping ribosome scanning, initiation, and reinitiation. By operating downstream of transcription, uORFs enable fine-scale control of protein abundance, offering a distinctive regulatory layer for precise tuning of gene expression in plants. Recent advances in ribosome profiling, translation initiation-site mapping, peptidomics, computational annotation, and genome editing have expanded the discovery, functional validation, and targeted manipulation of plant uORFs. In this review, we summarize current understanding of uORF-mediated translational regulation, discuss computational and experimental approaches for candidate identification and functional validation, and highlight genome-editing strategies used to modify endogenous uORFs in crops. We further synthesize representative studies in which uORF editing has been used to modulate metabolic traits, plant development, nutritional quality, and responses to biotic and abiotic stresses. Collectively, available evidence suggests that uORF manipulation can support quantitative and context-dependent trait modulation, in some cases preserving basal gene function and reducing growth fitness penalties. We further discuss outstanding challenges, including transcript isoform complexity, context-dependent translational effects, and the need for validation across diverse genetic and environmental backgrounds. Overall, uORF engineering represents a promising strategy for translational fine-tuning of plant gene expression, with potential implications for crop improvement and sustainable agriculture.

## 1 Introduction

Global agriculture faces mounting pressures from population growth, shrinking arable land, and accelerating climate change. Conventional breeding approaches based on phenotypic selection are increasingly insufficient to meet rapidly evolving consumer demands and environmental constraints. In the context of labor shortages and constrained agricultural supply, the urgency to enhance crop yield, quality, and stress resilience has intensified (Godfray et al., 2010; Bailey-Serres et al., 2019). In response, advances in genetic engineering, particularly genome editing, have provided powerful tools for crop improvement (Zhu et al., 2020; Puchta et al., 2022). These technologies enable precise modulation of gene expression, traditionally at the transcriptional level. However, because transcriptional regulation primarily governs mRNA abundance, additional regulatory layers are often required to achieve faster, reversible, and more finely graded control of protein synthesis in response to developmental and environmental signals (Gebauer and Hentze, 2004; Sonenberg and Hinnebusch, 2009).

Translational regulation offers a complementary and often more immediate means of modulating gene expression at the final step of protein production. This mode of control allows rapid responsiveness to environmental cues and supports subtle, graded adjustments in protein output (Hinnebusch et al., 2016; Merchante et al., 2017). uORFs, which are widespread and well-characterized regulatory elements located in the 5′UTR of mRNAs, play a central role in this process (Calvo et al., 2009). By influencing ribosome scanning and initiation, uORFs frequently suppress translation of downstream mORFs. Importantly, some uORFs act as signal-responsive modules whose inhibitory effects can be either alleviated or reinforced by metabolites or stress signals, thereby enabling rapid remodeling of protein output without requiring *de novo* transcription (Vattem and Wek, 2004; Rahmani et al., 2009; von Arnim et al., 2014).

With ongoing advances in CRISPR-Cas technologies, uORFs have emerged as key regulatory elements for precision crop breeding. Targeted editing of endogenous uORFs has successfully fine-tuned expression of genes underlying critical agronomic traits, associated with enhanced disease resistance (Tian et al., 2024), increased vitamin C accumulation in lettuce (Zhang et al., 2018), and optimized flowering time (Liu et al., 2021). Collectively, these studies indicate that uORF editing can provide graded control of gene expression while reducing the risk of severe pleiotropic effects often associated with coding-sequence knockouts. In parallel, computational approaches are beginning to expand the toolkit for large-scale identification of functional uORFs and for data-informed engineering of translational control elements. Together with advances in artificial intelligence (AI) and precision genome editing, these advances are making it increasingly feasible to identify, design and implement *de novo* or artificial uORFs for data-informed modulation of metabolic and developmental pathways (McGillivray et al., 2018; Xue et al., 2023).

In this review, we integrate recent advances in the molecular mechanisms of uORF-mediated regulation with current progress in uORF identification, validation, and genome editing in plants. We discuss the potential of uORFs as translational regulatory elements for crop improvement, while also emphasizing current limitations in predictability, mechanistic interpretation, and field-level validation. We hope this review provides a balanced framework for understanding both the promise and the present constraints of uORF engineering in plant biology and crop improvement.

## 2 Discovery, classification, and mechanisms of uORF-mediated translational regulation

### 2.1 Discovery and mechanistic models of uORF-mediated regulation

Eukaryotic gene expression is regulated across multiple layers, including transcriptional, translational, and post-transcriptional/post-translational processes. Among these, translation initiation has emerged as a dominant control point governing protein output while enabling rapid responses to developmental and environmental signals (Wethmar, 2014). Within this regulatory architecture, uORFs constitute one of the most influential cis-acting elements embedded in the 5′UTR. By modulating ribosomal scanning dynamics and start-codon recognition, uORFs exert strong, quantitative control over downstream mORF translation, thereby shaping protein synthesis with high precision.

The conceptual framework for uORF-mediated translational regulation was established through classic studies in yeast, most notably of the amino acid starvation regulator *GCN4*. The 5′UTR of *GCN4* mRNA contains four uORFs that coordinately govern ribosomal reinitiation, permitting selective translational induction of *GCN4* mRNA under amino acid limitation and forming an efficient translational circuit for nutrient stress adaptation (Hinnebusch, 2005). Analogously, in animals, the uORF architecture of *ATF4* functions as a central integrator of nutrient status and cellular stress. The 5′UTR of *ATF4* mRNA harbors two conserved uORFs: under basal conditions, ribosomes translate uORF1 and efficiently reinitiate at uORF2, which overlaps the main coding sequence, thereby suppressing mORF translation and limiting ATF4 protein accumulation. During amino acid deprivation, endoplasmic reticulum stress, or stress conditions, phosphorylation of eIF2α reduces the availability of initiation complexes, lowering the probability of reinitiation at uORF2 and allowing ribosomes to bypass it and initiate at the mORF, resulting in stress-induced ATF4 protein expression (Harding et al., 2000). In plants, uORF-dependent translational control has also been mechanistically elucidated. For example, conserved uORFs in the 5′UTRs of Arabidopsis bZIP transcription factor genes (e.g., *AtbZIP11*) encode short nascent peptides that induce ribosome stalling in response to elevated sucrose levels, thereby blocking downstream mORF translation and establishing a direct sucrose-sensing translational switch (Rook et al., 1998; Wiese et al., 2004; Rahmani et al., 2009). Collectively, these findings—from early forward genetic screens to detailed mechanistic analyses—demonstrate that uORFs are widely deployed as regulatory elements that couple environmental signals to protein output across eukaryotes.

The advent of ribosome profiling (Ribo-seq), proteomics, and advanced computational pipelines has enabled systematic, genome-wide discovery and annotation of actively translated uORFs. By sequencing ribosome-protected fragments (RPFs), Ribo-seq maps ribosome occupancy and pausing events at near-nucleotide resolution, providing strong evidence for uORF translation and functional relevance. In Arabidopsis, super-resolution ribosome profiling combined with transcriptome assembly identified 6,996 translated uORFs in protein-coding genes, revealing that uORF translation is far more prevalent than previously inferred from conventional annotation. Notably, approximately half of these uORFs encode peptides ≤10 amino acids, and their repressive effects on downstream translation do not follow a simple length-dependent relationship. Genome-wide analyses further show that transcripts harboring multiple translated uORFs exhibit reduced mORF translation efficiency (Wu et al., 2024). Comparable patterns have been observed in major crops. In rice, multi-tissue ribosome profiling identified 3,392 translated uORFs and showed that translational profiles vary more strongly among tissues than among varieties (Zhu et al., 2022). In addition, uORF-dependent regulation can respond to nutrient status, as exemplified by the phosphate-responsive control of *OsNLA1* expression (Yang et al., 2020), and global translational reprogramming is rapidly induced under salinity stress (Yang et al., 2021; Li et al., 2018). In maize, rare variants within 5′UTR uORFs alter downstream protein abundance, thereby shaping metabolism and physiological traits (Gage et al., 2022). Together, these studies establish uORF translation as a pervasive and highly plastic regulatory layer in plants and suggest that uORF sequence variation has contributed to adaptive evolution, providing a valuable substrate for precision modulation of gene expression in crop improvement.

### 2.2 uORF types and their mechanisms in controlling translation initiation

The regulatory influence of uORFs is strongly determined by their spatial configuration relative to the downstream mORF. Based on the position of the uORF stop codon and its reading frame relative to the mORF start site, uORFs can be classified into four principal categories: (i) non-overlapping uORFs, whose stop codon lies upstream of the mORF start codon; (ii) out-of-frame overlapping uORFs, whose stop codon resides within the mORF coding region in a different reading frame; (iii) in-frame overlapping uORFs, which share the same reading frame as the mORF and often terminate at the same stop codon (Zhang et al., 2020; Liu et al., 2023); and (iv) compound uORFs, in which multiple uORFs of different classes coexist within a single 5′UTR (Figure 1A). This classification captures structural diversity and provides a mechanistic framework for understanding variation in repression strength among distinct uORF architectures (Mou et al., 2025).

**FIGURE 1:**
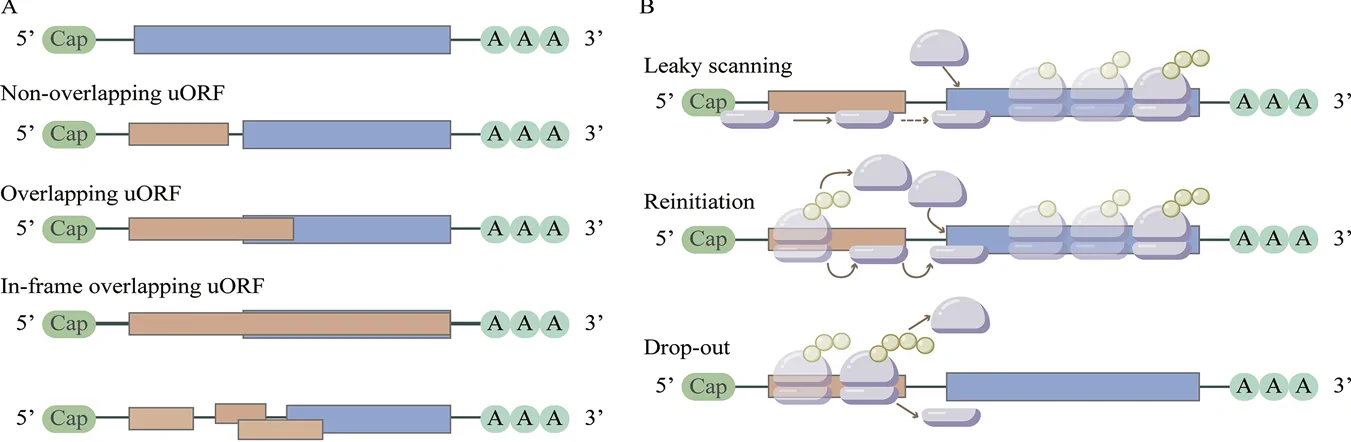
Structural diversity and translational regulatory mechanisms of upstream open reading frames (uORFs) in plant mRNAs. **(A)** Schematic representation of distinct uORF architectures within the 5' untranslated region (5'UTR) of plant mRNAs. uORFs are classified based on their positional and reading–frame relationships with the main open reading frame (mORF), including non–overlapping uORFs, overlapping uORFs, in–frame overlapping uORFs, and multi–uORF configurations. These structural features critically shape ribosome behavior and downstream translational output. **(B)** Major mechanisms by which uORFs modulate translation initiation and efficiency of the mORF. Following cap–dependent recruitment, scanning ribosomes may initiate at a uORF start codon, leading to (i) ribosome reinitiation after uORF termination, depending on uORF length and intercistronic distance; (ii) leaky scanning, allowing ribosomes to bypass weak uORF start codons and initiate at the mORF; or (iii) ribosome stalling and premature dissociation, resulting in translational repression of the mORF. In some cases, uORF–encoded peptides or specific sequence contexts promote ribosome stalling, acting as regulatory checkpoints responsive to cellular or environmental cues. Together, these mechanisms establish uORFs as key cis–regulatory elements that fine–tune gene expression at the translational level.

In the canonical scanning model of eukaryotic translation initiation, the 43 S pre-initiation complex (PIC) assembles at the 5’cap and scans in the 5'→3′direction until it encounters an AUG start codon in a favorable initiation context. Because of their upstream location, uORFs are often encountered before the mORF and thus reduce the probability that scanning ribosomes will initiate at the downstream start site, leading to translational repression. When the PIC encounters a canonical uORF (initiated at AUG and terminated at UAA, UAG, or UGA), at least three outcomes are possible (Figure 1B). First, leaky scanning may occur when the uAUG resides in a weak Kozak context, allowing a fraction of scanning complexes to bypass the uAUG and initiate at the mORF, thereby sustaining basal translation under non-stress conditions. Second, reinitiation may occur after uORF translation: following termination, the 40 S subunit can remain associated with the mRNA, retain a subset of initiation factors, resume scanning, and reinitiate at the mORF. Third, ribosomes may stall near the uORF termination region, triggering nonsense-mediated mRNA decay (NMD), or both ribosomal subunits may dissociate from the mRNA, terminating further translation (Kozak, 1981; 1989; Hinnebusch, 2014; Schleich et al., 2014; Uchiyama-Kadokura et al., 2014; Zhang et al., 2019).

The extent of uORF-mediated repression is further shaped by multiple parameters, including uORF number, length, start-codon context strength, and spacing between adjacent uORFs. In general, long uORFs, uORFs encoding peptides that promote ribosome stalling, or 5′UTRs containing multiple sequential uORFs severely compromise reinitiation, favor ribosome stalling and dissociation, and impose near-complete translational blockade of the downstream mORF (Wethmar, 2014; Gunišová et al., 2018; Zhang et al., 2019). Ribosomal outcomes are also tightly constrained by uORF architecture. For non-overlapping uORFs, whose stop codons precede the mORF start site, downstream translation can, in principle, proceed via either leaky scanning or reinitiation. In contrast, for overlapping uORFs whose stop codons fall within the mORF coding region, termination occurs internally within the mORF, making conventional reinitiation at the mORF start site unlikely. Consequently, mORF translation depends largely on leaky scanning, an inherently inefficient initiation route, rendering overlapping uORFs generally more stringent and difficult-to-bypass repressors of downstream translation (Hinnebusch, 2014; Hinnebusch et al., 2016).

## 3 Frameworks for uORF identification, characterization, and functional validation

### 3.1 Bioinformatic strategies for uORF candidate identification

Bioinformatic approaches constitute a major entry point for the genome-scale identification of candidate uORFs in plants and are increasingly evolving from linear sequence-rule scanning toward more integrative frameworks that incorporate evolutionary, structural, and translational-context features (Whiffin et al., 2020). As a general annotation principle, a uORF is typically defined as the presence of an initiation codon in an annotated mRNA 5′UTR and can be further classified according to the position of its stop codon relative to the mORF (Niu et al., 2020). Importantly, uORF annotation is inherently transcript dependent rather than gene dependent, since alternative TISs, TSS choice, and transcript isoform diversity can determine whether a given uORF is present at all (Li and Liu, 2020; Kurihara et al., 2018). In addition, accumulating evidence indicates that non-AUG-initiated uORFs can also exert biologically meaningful regulatory effects (Spealman et al., 2018). In complex plant transcriptomes, thousands of annotated uORFs have been identified, yet only a fraction exerts measurable effects on downstream translation or conditional gene expression.

Among the large number of annotated uORFs in plant transcriptomes, a subset shows evolutionary conservation across species, making comparative analysis an important strategy for identifying potentially functional candidates. In line with this, a range of bioinformatic approaches has been developed to identify candidate regulatory uORFs on the basis of evolutionary conservation or sequence homology to previously characterized uORFs. Early comparisons between Arabidopsis and rice identified multiple conserved uORF families with signatures of purifying selection, and pipelines such as ESUCA have expanded CPuORF across angiosperms (Hayden and Jorgensen, 2007; Takahashi et al., 2020). At the same time, accumulating evidence suggests that many uORFs have emerged relatively recently in evolution, and that some are conserved only within particular families or narrower phylogenetic groups (Sandmann et al., 2023).

Dedicated databases provide practical resources for retrieving candidate uORFs and related small ORFs. PsORF (https://psorf.whu.edu.cn/) serves as a plant-focused resource for sORF discovery, cataloging candidates from 5′UTRs, 3′UTRs, and non-coding transcripts, with support from genomic annotation, transcriptomic data, Ribo-seq, and mass spectrometry evidence (Chen et al., 2020). uORFlight (http://www.rnairport.com:443/) provides a variation-centered platform for plant uORF analysis in Arabidopsis and rice, integrating alternative splicing models, population-level sequence variation, and potential links between uORF variants and phenotypic traits (Niu et al., 2020). uORFdb (https://www.bioinformatics.uni-muenster.de/tools/uorfdb/index.pl) offers a broader annotation framework by integrating curated uORF information, sequence context, transcript annotation, and variation data across multiple eukaryotic species (Manske et al., 2023). Ribo-uORF (https://rnainformatics.org.cn/RiboUORF/) adds translation-level evidence through ribosome profiling and initiation-site profiling data for translated uORFs and upstream initiation events in model organisms (Liu et al., 2023). Collectively, these resources support candidate screening, annotation, and hypothesis generation (Table 1).

**TABLE 1:** Public resources useful for uORF/sORF discovery and prioritization.

Tool	Url	Main content	Use in uORF analysis	References
PsORF	https://psorf.whu.edu.cn/	Plant sORFs from 35 species; includes uORFs, uoORFs, dORFs, doORFs and other sORFs	Screening plant sORF/uORF candidates with multi-omics support	Chen et al. (2020)
uORFlight	http://uorflight.whu.edu.cn	uORF variation in arabidopsis and rice; splicing models, SNPs, INDELs and trait-related information	Prioritizing plant uORFs with potential regulatory or phenotypic relevance	Niu et al. (2020)
uORFdb	https://www.bioinformatics.uni-muenster.de/tools/uorfdb/index.pl	Curated uORF information, sequence context, transcript annotation and variation data	Retrieving known and predicted uORFs across eukaryotic species	Manske et al. (2023)
Ribo-uORF	https://rnainformatics.org.cn/RiboUORF/	Translated uORFs and upstream translation initiation sites from ribo-seq and QTI-seq data	Providing methodological reference for ribosome-supported uORF annotation	Liu et al. (2023)

### 3.2 Experimental approaches for evaluating plant uORF activity

Following candidate identification, experimental evaluation of plant uORFs focuses on determining whether candidate uORFs are translated, produce detectable peptide products, and influence downstream gene expression. The key question is not simply whether a uORF is present in a transcript, but whether it is actively engaged under relevant biological conditions and contributes to gene regulation in a measurable way.

Ribosome profiling and translation initiation-site profiling are widely used to determine whether candidate uORFs are actively translated (Ingolia et al., 2009; Lee et al., 2012). Ribosome footprint enrichment, reading-frame preference, and 3-nt periodicity are key indicators of translated uORFs (Juntawong et al., 2014; Hsu et al., 2016; Wu et al., 2024). In Arabidopsis, these approaches revealed widespread translation in 5′UTRs and supported uORF identification (Juntawong et al., 2014). Combined with RNA-seq and structural prediction, Ribo-seq can further assess how RNA folding, initiation-codon context, and translational efficiency affect uORF translation (Kim et al., 2014; Xiang et al., 2023; Wu and Hsu, 2024). At the quantitative level, translational efficiency (TE) remains widely used, whereas RuM may provide complementary insight into the translational relationship between a uORF and its associated mORF (Wu and Hsu, 2024). Together, these approaches are especially valuable for defining the translational status of candidate uORFs across tissues, genotypes, and conditions.

Complementing translation-based evidence, peptidomics, a mass spectrometry (MS)-based high-throughput approach, provides an important means of detecting peptide products derived from candidate uORFs. In plants, this strategy has facilitated the discovery of diverse endogenous peptides involved in stress responses, immunity, and developmental regulation. For example, genome-scale profiling of endogenous peptides in grape suggested that non-AUG-initiated translation is widespread (Pei et al., 2022). Similarly, deep peptidomic analysis across 13 maize tissues identified 6,100 non-redundant endogenous peptides (Ali et al., 2024). More recently, a uORF-encoded mobile peptide was reported to trigger systemic stomatal immunity, further supporting the view that uORF translation can give rise to biologically active peptide products (Liu et al., 2026).

To experimentally evaluate the relative effects of sequence, structural, and positional features on uORF activity, transient reporter assays are commonly used to associate these features with changes in protein expression. For instance, in yeast, the high-throughput transient reporter systems FACS-uORF and PoLib-seq have been applied to quantify the regulatory activity of uORFs (Gemma et al., 2023). In plants, dual-luciferase transient assay systems have been used to examine uORF-mediated translational repression (Wu et al., 2022). This layer establishes a relationship between uORF translation and downstream.

In summary, uORF activity cannot be inferred from sequence annotation alone, its evaluation usually requires multiple layers of evidence, including sequence-based prioritization, ribosome-based translation signals, peptide detection, and reporter-based functional validation (Table 2). These readouts should be interpreted as complementary evidence rather than fixed universal thresholds, their values may vary across species, tissues, conditions, and analytical pipelines.

**TABLE 2:** Representative readouts for functional uORF prediction and evaluation.

Evidence type	Key readouts	Main use	References
Sequence-based prioritization	Conservation, dN/dS, start-codon context, RNA structure	Candidate screening	Lin et al., 2011; Kim et al., 2014; Xiang et al., 2023
Ribosome-based evidence	Ribosome footprints, 3-nt periodicity, reading frame, TIS signal	Translation evidence	Ingolia et al., 2009; Lee et al., 2012; Juntawong et al., 2014; Hsu et al., 2016
Translational relationship	TE, uORF/mORF ribosome occupancy, RuM	uORF–mORF regulation	Wu and Hsu (2024)
Peptide-level evidence	MS-detected peptides, peptide abundance	Peptide production	Pei et al., 2022; Ali et al., 2024; Liu et al., 2026
Reporter-based validation	Luciferase ratio, protein/mRNA output, uAUG mutation	Functional effect	Wu et al., 2022; Gemma E et al., 2023

### 3.3 Gene-editing frameworks and strategy selection for functional validation of uORF activity

Following computational prioritization of translationally active uORFs, genetic manipulation is essential for establishing their *bona fide* biological relevance *in vivo.* Although mechanistic studies have frequently relied on transient reporter assays, functional validation and crop deployment increasingly depend on editing endogenous uORFs in their native genomic context. Currently, CRISPR/Cas9 and its derived systems are the dominant technologies for this purpose.

Current engineering strategies address two primary objectives: (i) removal of repressive uORFs to derepress beneficial genes, and (ii) rational rewiring or installation of synthetic uORFs to implement conditional translational control. These complementary strategies have been successfully applied across multiple crop species, enabling decoupling of resistance or tolerance traits from growth penalties. Based on target parameters—including start codon identity, Kozak strength, termination context, and steric relationships to the mORF—three genome-editing tiers form an integrated technical portfolio.

At the foundational tier, CRISPR/Cas9-mediated gene knockout (Cas9-KO) is a robust technology that utilizes single-guide RNAs (sgRNAs) to direct the Cas9 nuclease to specific genomic loci, introducing DNA double-strand breaks (DSBs) that are predominantly repaired by non-homologous end joining (NHEJ) to generate localized insertions or deletions (indels) (Jinek et al., 2012; Cong et al., 2013). In practice, sgRNAs are preferentially designed to target the start codon of the target uORF, subsequent NHEJ-induced indels structurally disrupt the uORF by preventing translation initiation, shifting the reading frame, or introducing premature stop codons (Figure 2A). Importantly, for genes harboring multiple uORFs within a single 5′leader, CRISPR/Cas9 enables the individual targeting and editing of each distinct uORF, allowing to screen the “major-effect” uORF that dominantly dictates translational repression. Leveraging this mechanism, CRISPR/Cas9-mediated uORF editing has been successfully applied to generate transgene-free crops with enhanced agronomic and nutritional traits across various species, including Arabidopsis, lettuce, rice and tomato (Zhang et al., 2018; Zhang et al., 2025; Lu et al., 2024). While highly efficient, this traditional deletion strategy carries a potential risk of inducing larger unintended deletions or rearrangements in the 5′leader sequence.

**FIGURE 2:**
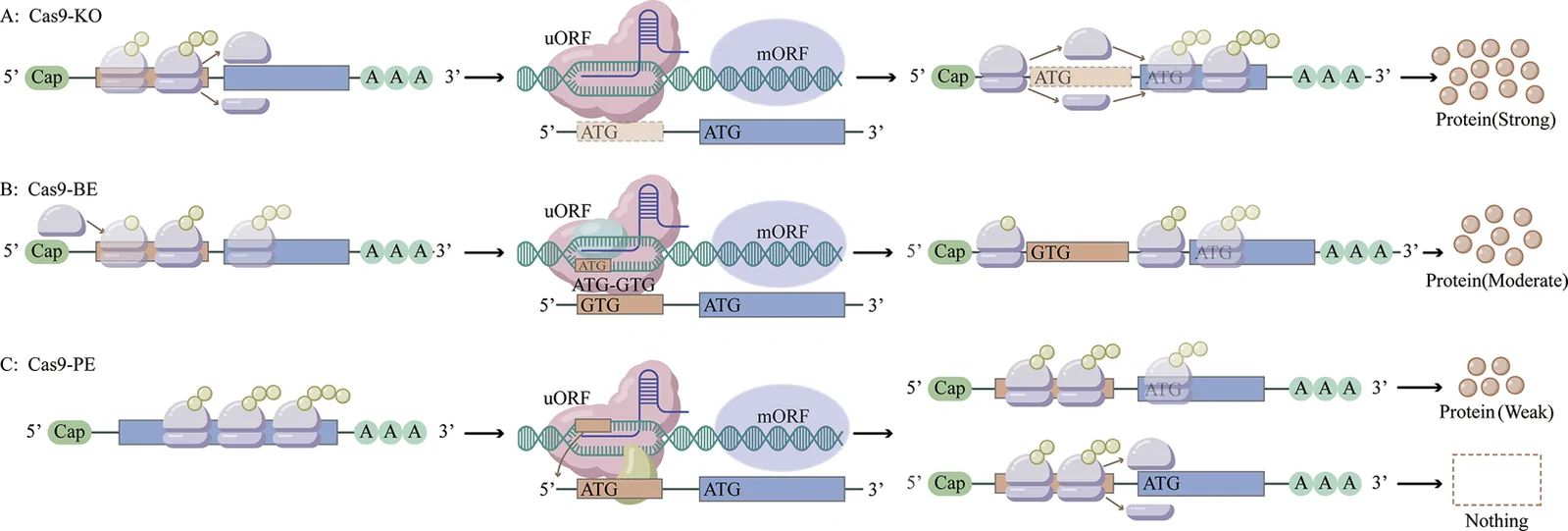
Tunable post–transcriptional regulation using CRISPR–based uORF engineering toolkits. **(A)** CRISPR–Cas9–mediated elimination of inhibitory uORFs fully activates mORF translation, generating corresponding phenotypic outputs (top right). **(B)** Base–editing–mediated modification of uORF start codons enables quantitative tuning of mORF expression: conversion of the initiating ATG to the non–canonical GTG yields a moderately reduced protein output. (C) Prime–editing–dependent uORF insertion creates complex translational control circuits in naïve gene architectures: weak constitutive uORFs support muted basal mORF expression (left), whereas strong repressive uORFs fully block output under specific cellular contexts (right).

To mitigate such risks and achieve more refined control, Base editing (BE) technologies, including cytosine and adenine base editors (CBEs and ABEs), enable precise and targeted nucleotide substitutions (such as C-to-T or A-to-G conversions) without generating DNA DSBs or requiring donor templates (Komor et al., 2016; Gaudelli et al., 2017; Rees and Liu, 2018). By employing BEs, researchers can precisely manipulate upstream uORFs to bidirectionally tune the translation of downstream mORFs. Specifically, this precision editing can completely abolish translation initiation by mutating an endogenous start codon (e.g., ATG to GTG), extend an existing uORF by disrupting its stop codon (e.g., TAG to TGG), create *de novo* uORFs by generating novel start codons (e.g., ACG to ATG), or alter the suppressive strength of a uORF by introducing missense mutations within highly conserved peptide motifs (Figure 2B). By leveraging base editors to precisely mutate, switch, or *de novo* create uORFs, this technology has been successfully applied to fine-tune diverse crop traits, exemplified by the targeted editing of *FvebZIPs1.1* in strawberry to optimize sugar content, the manipulation of *OsGS2* in rice to balance broad-spectrum disease resistance, and the modification of *OsDLT* in rice to achieve the graded downregulation of plant architecture (Xing et al., 2020; Xue et al., 2023; Mou et al., 2025).

Prime editing (PE), a highly versatile CRISPR-derived technology, directly rewrites target genomic sequences by combining a Cas9 nickase, a reverse transcriptase, and a prime editing guide RNA (pegRNA), thereby enabling precise nucleotide substitutions, insertions, and deletions (Anzalone et al., 2019; Lin et al., 2020). This capability uniquely positions PE for sophisticated uORF engineering, where translational regulatory strength often depends on complex structural features—such as ORF length and intercistronic spacing—rather than merely on single-base changes. Unlike base editors, which are constrained by specific editing windows and targetable base transitions, PE facilitates the construction of entirely novel regulatory architectures. Through programmable sequence writing, researchers can systematically generate *de novo* uORFs, extend existing ones, or optimize the spatial dynamics between uORFs and the downstream mORF (Figure 2C). The remarkable utility of this approach was recently demonstrated in rice, where PE-mediated structural rewriting of endogenous 5′UTRs enabled the creation of bespoke uORFs, thereby achieving the precise, graded downregulation of critical agronomic traits (Xue et al., 2023).

## 4 Applications of uORF editing strategies in crop trait improvement

As understanding of uORF-mediated translational control has progressed from mechanistic dissection to precise genome engineering, uORFs have emerged as tractable targets for crop improvement. A common feature across current applications is that uORF editing rarely acts as a simple on/off switch. Instead, by altering ribosome access to downstream coding sequences, it enables graded adjustment of endogenous gene output. This quantitative mode of regulation is particularly valuable for agronomic traits that depend on dosage-sensitive expression, including metabolic composition, developmental transitions, immune activation, and abiotic stress responses (Table 3).

**TABLE 3:** Representative Applications of uORF Editing Strategies for Crop Trait Improvement.

Species	Target gene	Target trait	Editing strategy and mechanism	Core tools	Major outcomes	References
Rice	*OsDLT*	Plant architecture	Extension of endogenous uORF and adjustment of intercistronic region	CRISPR-PE/BE	Graded downregulation of brassinosteroid signaling, generation of allelic series with continuous architectural phenotypes, and demonstration of uORFs as molecular dimmers	Xue et al. (2023)
​	*OsBRI1*	Compact architecture	Generation of *de novo* uORF in the 5′UTR	​	Introduction of a translational repressor element, suppression of pORF translation, and production of compact plant stature with reduced BR responsiveness	​
​	*OsGS2*	Disease resistance	Switching uORF type in the 5′leader	CRISPR-BE	Transgene-free broad-spectrum pathogen resistance with minimal growth penalty and high transferability across crop species	Tian et al. (2024)
​	*OsHD2*	Flowering time	Editing uORFs in the 5′UTR	CRISPR-INDEL/BE	Efficient and mild flowering delay achieved through translational derepression without transcriptional changes, saving time and labor relative to conventional breeding	Liu et al. (2021)
​	*OsWaxy*	Amylose content	Editing *Wx* uORF6	CRISPR-BE	Significant reduction in amylose content, yielding grains with improved texture and semi-translucent appearance	Lu et al. (2024)
​	*OsGLW7*	Grain shape and yield	Disruption of repressive uORFs	CRISPR-INDEL	Markedly longer grains with significantly increased length-to-width ratio and up to 19.4% higher thousand-grain weight	Yang et al., 2025
​	*OsTBF1*	Disease resistance	Truncation of the *OsTBF1* uORF	CRISPR-INDEL	Increased OsTBF1 protein accumulation and enhanced disease resistance without growth suppression	Tian et al., 2025
​	*OsABA2*	Pre-harvest sprouting	Disruption of the *OsABA2* uORF	CRISPR-INDEL	Moderate increases in OsABA2 protein levels and ABA accumulation, significantly enhancing seed dormancy while avoiding growth penalties	Wang et al. (2025)
Lettuce	*LsGGP2*	Vitamin C content	Mutation of the uORF initiation region	CRISPR-INDEL	Markedly increased translational efficiency and ∼150% elevation in leaf vitamin C content, demonstrating an effective nutritional enhancement strategy	Zhang et al. (2018)
Tomato	*SlRabGAP22*	Salt tolerance	Editing functional uORFs in the 5′leader	CRISPR/Cas9	First demonstration of improved salt tolerance in tomato via uORF editing, establishing a new approach for stress-resilient breeding	Jia et al. (2024)
​	*SlTDC1*	Serotonin content	Editing three uORFs upstream of *SlTDC1*	CRISPR/Cas9	Selective enhancement of serotonin biosynthesis without adverse effects on plant growth	Zhang et al. (2025)
Canola	*BnVTC2*	Vitamin C and stress tolerance	Editing *BnVTC2* uORFs	CRISPR-INDEL	Concurrent improvement of nutritional value and abiotic stress tolerance without yield reduction, establishing uORF editing as a novel avenue for crop improvement	Hao et al. (2025)

### 4.1 Stepwise uORF-mediated regulation of metabolic pathways for crop quality improvement

uORFs are particularly well suited for engineering metabolic traits because desirable phenotypes in this category often depend on quantitative changes in pathway flux. By modulating translation of endogenous biosynthetic genes, uORF editing provides a means of adjusting protein output while preserving the broader structure of the pathway. Current studies highlight two main applications of this strategy: removal of endogenous inhibitory uORFs to increase beneficial metabolites, and introduction of synthetic upstream elements to suppress undesirable quality traits. In lettuce, editing the uORF of *LsGGP2*, which encodes a key enzyme in vitamin C biosynthesis, was associated with ∼150% higher ascorbate content and increased oxidative stress tolerance (Zhang et al., 2018). A comparable approach was reported in tomato, where editing three uORFs in the upstream regulatory region of *SlTDC1* elevated serotonin accumulation in ripening fruits by 1.8- to 3.1-fold (Zhang et al., 2025). uORF engineering can also be used in the opposite direction. In rice, targeted insertion of a synthetic upstream ATG-containing element into the 5′UTR of *Waxy* reduced amylose-associated grain quality traits, showing that translational repression can be rationally introduced into endogenous quality genes (Shen et al., 2024). These examples illustrate how uORF editing can be used to reshape metabolic traits in a stepwise manner, with clear potential to improve both nutritional value and eating quality through precise modification of upstream cis-regulatory elements.

### 4.2 Fine-tuning key gene dosage via uORFs to modulate development and yield

Compared with metabolic traits, developmental and yield-related phenotypes are often governed by regulatory genes whose effects are strongly dosage dependent. For these targets, complete loss-of-function alleles are frequently too severe to be agronomically useful, whereas uORF editing can produce more moderate phenotypic shifts. This has now been demonstrated across several developmental traits, including flowering time, seed behavior, and plant architecture. In rice, editing the uORFs in the 5′leader of *Hd2*, a flowering repressor in the photoperiod pathway, generated homozygous lines that headed 4.6–11.2 days later than the wild type. This phenotype was accompanied by reduced expression of the downstream flowering regulators *Ehd1*, *Hd3a*, and *RFT1* (Liu et al., 2021). In hormone-mediated seed developmental transitions, *ABA2/OsABA2* in Arabidopsis and rice contains a conserved uORF that represses translation of the downstream coding sequence. In rice, disruption of the *OsABA2* uORF was associated with reduced preharvest sprouting, and natural variation in the same region was linked to differences in *OsABA2* expression and sprouting behavior among cultivars (Wang et al., 2025). A similar principle also applies to rice architecture-related regulators, engineered *de novo* uORFs or extended endogenous uORFs in *OsDLT*, a GRAS-family component of brassinosteroid signaling, generated a series of lines with progressively altered plant height, lamina inclination and tiller number (Xue et al., 2023). Together, these studies show that uORF manipulation provides a practical route to optimize developmental-related traits by quantitatively adjusting the expression of key endogenous regulators.

### 4.3 Precision strategies for achieving disease resistance without growth penalties

A major challenge in disease-resistance engineering is to enhance immunity without causing constitutive growth penalties. uORFs provide an attractive solution because they can restrict defense protein accumulation under normal conditions while permitting rapid activation upon pathogen challenge. This principle was first demonstrated using a *TBF1*-based regulatory cassette, in which an immune-inducible promoter and two pathogen-responsive uORFs were used to control *snc1* in Arabidopsis and *AtNPR1* in rice, thereby improving broad-spectrum disease resistance while greatly reducing the developmental costs associated with constitutive immune activation (Xu et al., 2017). More recently, uORF engineering has been extended to endogenous crop genes. In rice, CRISPR-aTrE-uORF editing identified an *OsGS2* allele in which conversion of a Type1 uORF to a Type2 uORF enhanced translational repression and contributed resistance to bacterial blight, bacterial leaf streak and blast with only limited fitness, whereas complete knockout caused severe growth defects (Tian et al., 2024). Beyond translational gating, uORFs can also encode bioactive immune signals, the *MYB51*-associated uORF was recently shown to produce the mobile peptide, which mediates systemic stomatal immunity through the SIRK1-KIN7 receptor complex in *Arabidopsis*, expanding the functional scope of uORFs from cis-regulatory elements to signaling molecules with potential relevance for future engineering (Liu et al., 2026). Together, these studies show that uORFs can be exploited at multiple levels to improve disease resistance while minimizing growth-defense trade-offs.

### 4.4 uORF-mediated responsive translational control of crop nutrition and abiotic stress adaptation

uORF engineering is also increasingly being used to improve plant resilience under adverse environmental conditions, both by enhancing direct abiotic stress tolerance and by preserving defense functions that are otherwise weakened under stress. In Arabidopsis, 35 S:uORFs_TBF1_-CBP60 g maintained salicylic acid-associated resistance at elevated temperature while avoiding the growth and flowering defects caused by constitutive *CBP60g* overexpression, showing that uORF-mediated translational control can buffer climate-associated suppression of immunity without aggravating growth-defense trade-offs (Kim et al., 2022). A more direct application to abiotic stress adaptation has been demonstrated in genes linked to antioxidant metabolism and stress-responsive regulation. In rice, editing the endogenous uORF of *OsGGP*, which encodes a rate-limiting enzyme in ascorbate biosynthesis, increased ascorbic acid accumulation and improved osmotic stress tolerance (Sang et al., 2025). In tomato, CRISPR/Cas9-mediated editing of multiple uORFs in the 5′leader of *SlRabGAP22* enhanced salt tolerance through improved reactive oxygen species scavenging, reduced Na^+^ toxicity, and alleviated osmotic stress effects (Jia et al., 2024). Similarly, editing the *BnVTC2* uORF in oilseed rape increased ascorbic acid accumulation and enhanced tolerance to low temperature, salinity, and drought without obvious yield penalties, generating favorable regulatory variation absent from natural germplasm (Hao et al., 2025). Together, these studies indicate that engineered uORFs provide versatile targets for strengthening abiotic stress resilience.

Across these applications, a common theme is that uORF engineering provides a form of translational dosage control that is often better suited to complex agronomic traits than either constitutive overexpression or complete gene disruption. By acting on endogenous regulatory elements, this strategy can preserve native expression contexts while still shifting protein output enough to alter phenotype. In practice, this allows uORF manipulation to resolve several recurrent breeding dilemmas, such as improving resistance without growth penalties, modifying development without severe pleiotropy, or enhancing stress tolerance while retaining yield performance. More broadly, these studies position uORF editing as a distinct and increasingly versatile branch of precision breeding, one that complements promoter editing and coding-sequence engineering by targeting the translational layer of gene regulation. Future progress will likely depend on more systematic identification of functional uORFs, clearer predictive rules linking uORF architecture to translational output, and expanded validation across diverse crop species and trait categories.

## 5 Conclusions and future perspectives

uORFs have emerged as a widespread and versatile layer of translational regulation in plants. In this review, we summarized current progress in the identification, mechanistic classification, and engineering application of plant uORFs, highlighting their value as tunable cis-regulatory elements for quantitative control of endogenous gene expression. Unlike conventional knockout strategies or promoter engineering, uORF manipulation offers a distinctive route to modulate gene output at the translational level, making it particularly attractive for traits that require dosage sensitivity, conditional responsiveness, and reduced pleiotropic penalties (Figure 3).

**FIGURE 3:**
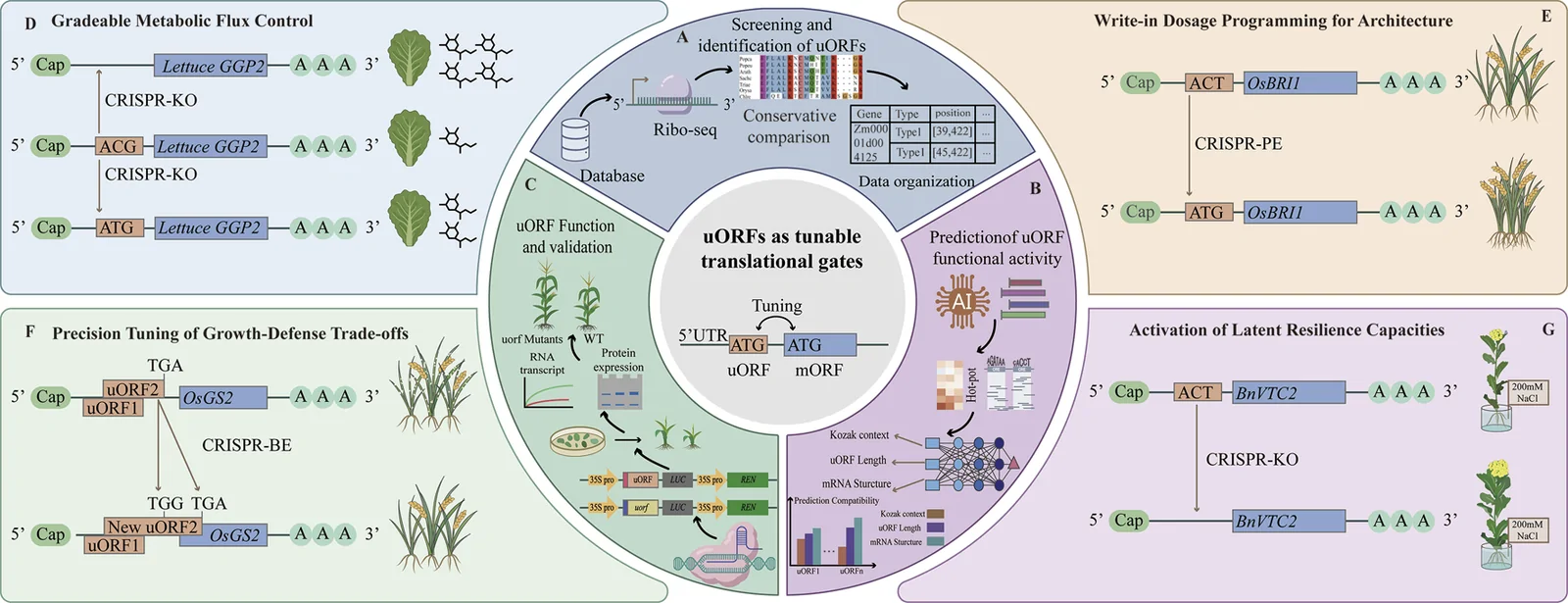
A Closed–Loop Paradigm for Precision Crop Engineering via uORF Reprogramming. The central workflow illustrates the transition from observation to predictive design. **(A-C)** The “Discovery–Prediction–Validation” cycle: novel 5' regulatory elements are identified via Ribo–seq and conservation filtering **(A)**, parameterized using deep–learning models that predict translational features (e.g., Kozak strength weighting) **(B)**, and experimentally validated at the protein level **(C)** before establishing trait applicability. **(D-G)** Diverse agronomic trait applications: **(D)** Graded metabolic rheostats --editing the start codon hierarchy of the lettuce LsGGP2 uORF enables stepwise tuning of vitamin C accumulation, with full uORF ablation (CRISPR–KO) maximizing flux and start codon conversion (ATG vs. ACG) enabling graded control without physiological penalties. **(E)** Architectural programming–Prime Editing (PE) introduces translational barriers (e.g., start codon insertion, ACT→ATG) upstream of dosage–sensitive regulators such as OsBRI1, converting tall phenotypes into lodging–resistant semi–dwarfs via cis–regulatory attenuation. **(F)** Growth–defense decoupling–Base Editing (BE) remodels the OsGS2 5' UTR array (e.g., stop–loss extension or creation of novel sorting uORFs), subtly reprogramming ribosomal scanning to prime standby immunity while preserving yield, thereby resolving classical fitness costs. **(G)** Latent stress unlocking–targeted removal (CRISPR–KO) of evolutionarily conserved “brake” motifs in stress regulators such as BnVTC2 acts as a stress–mimicking release switch, unleashing full antioxidative capacity and conferring salinity tolerance absent from wild–type alleles.

Addressing these challenges will require a shift from descriptive cataloging toward integrative and integrative and experimentally validated frameworks. In this context, artificial intelligence may provide an important enabling layer. Recent advances in machine learning, deep learning, and generative modeling have already begun to transform modern agricultural biotechnology by improving genomic prediction (Li et al., 2024), identifying regulatory elements across species (Li et al., 2026), and enabling synthetic promoter design for transcriptional control (Xiang et al., 2024). For uORF research, similar strategies could be used to integrate transcriptomic, translatomic, proteomic, metabolomic, and natural-variation datasets to identify functional uORFs, infer sequence–function relationships, prioritize editing targets, and guide the design of regulatory architectures with more interpretable translational outputs. AI-assisted models may help address the context dependence that has so far limited the portability of empirical design rules across genes, tissues, and species.

An especially important Frontier lies in metabolite-responsive uORFs, which bridge mechanistic insight and synthetic design. Although some studies suggest that uORF-encoded peptides can couple metabolite status to translational output (Mou et al., 2025; Zhang et al., 2020; Álvarez-Urdiola et al., 2025), how plant uORFs sense specific metabolites and how such mechanisms can be rationally redesigned remain largely unresolved. Resolving these questions will be essential not only for understanding endogenous translational control, but also for the *de novo* construction of synthetic translational sensors that link cellular metabolic states to agronomically important outputs.

Overall, future progress in plant uORF biology will depend on the convergence of improved annotation, mechanistic dissection, predictive modeling, and precision engineering. More broadly, uORF research is now moving from descriptive cataloging toward hypothesis-guided regulatory engineering. As functional maps and editing technologies continue to improve, uORFs may become an important framework for translational dosage control in next-generation precision breeding.
